# Degradation of Dye Wastewater by a Novel mBT-MPR Visible Light Photocatalytic System

**DOI:** 10.3390/ijerph20010571

**Published:** 2022-12-29

**Authors:** Miaomiao Cheng, Chunxia Zhao, Zefeng Wu, Ling Liu, Hongjie Wang

**Affiliations:** 1 Hebei Key Laboratory of Close-to-Nature Restoration Technology of Wetlands, School of Eco-Environment, Hebei University, Baoding 071002, China; 2 Institute of Xiong’an New Area, College of Life Science, Hebei University, Baoding 071002, China

**Keywords:** magnetic visible light photocatalyst, magnetic field controlled photocatalytic reactor, dye wastewater, rhodamine B

## Abstract

The high efficiency and low consumption green wastewater treatment technology has important practical significance for the recycling of printing and dyeing wastewater. The efficiency of visible light catalytic degradation of organics is greatly affected by the performance of the catalyst and the photo reactor. Therefore, Bi_2_WO_6_/TiO_2_/Fe_3_O_4_ (mBT) visible light photocatalyst was accurately prepared by the ammonia iron double drop method. In order to improve the photodegradation efficiency, a tubular magnetic field-controlled photocatalytic reactor (MPR) was developed. The novel mBT-MPR visible light photocatalytic system was proposed to treat RhB simulated wastewater. The experimental results showed that when the dosage of mBT catalyst was 1 g/L and visible light was irradiated for 60 min, the average removal rate of rhodamine B (RhB) with initial an concentration of 10 mg/L in the simulated wastewater for four times was 91.7%. The mBT-MPR visible light photocatalysis system is a green and efficient treatment technology for organic pollutants in water with simple operation, low energy consumption, and no need for catalyst separation.

## 1. Introduction

Various dyes and additives enter the surface water body due to the inadequate treatment of printing and dyeing wastewater, causing harm to aquatic ecology and human health. Therefore, it is particularly important to develop efficient, simple, and low-consumption treatment methods. The traditional printing and dyeing wastewater treatment methods mainly include physical treatment, chemical treatment, and biological treatment, but they all have problems of varying degrees, such as complex operation, high cost, and secondary pollution. In recent years, the application of photocatalysis technology to the degradation of pollutants has received extensive attention and can effectively alleviate environmental pollution [1,2,3]. Photocatalytic technology uses the energy of sunlight through various semiconductor materials, and the electrons and holes generated by it have strong reducibility and oxidizability, respectively [4,5,6,7].

Many photocatalysts with narrow band gaps have excellent photocatalytic activity under visible light irradiation, such as Bi_2_WO_6_ [8], BiOI [9], and g-C_3_N_4_ [10]. However, there are still some problems, such as the small response range of visible light and the high recombination rate of photogenerated carriers, which limit its application. Among these catalysts, Bi_2_WO_6_ (Eg = 2.7 eV) is the simplest perovskite sheet structure composed of a (Bi_2_O_2_)^2+^ layer and a (WO_4_)^2−^ layer arranged alternately [11]. Bi_2_WO_6_ has a unique layered structure and a suitable band gap structure, making it a photocatalyst with a good response to visible light [12]. Bi_2_WO_6_ has excellent physical and chemical properties such as ferroelectric voltage, catalytic behavior, and nonlinear dielectric susceptibility that are non-toxic and harmless and can be used for organic matter degradation and oxygen release [13,14]. However, the fast recombination speed of photogenerated carriers inhibits their light energy conversion efficiency, so it is necessary to ameliorate them. As one of the most widely studied metal oxides, titanium dioxide (TiO_2_) has the properties of relatively high quantum yield, low cost, easy to obtain and low toxicity, and is relatively stable under light. The biggest disadvantage is that only about 5% of the ultraviolet light can be used, and the utilization of solar light is very low.

Referring to the literature about Bi_2_WO_6_, there are two routes to improving the catalytic activity: expanding the light absorption range of the photocatalyst and improving the separation efficiency of photogenerated carriers. The specific methods are listed as follows: depositing precious metals on the surface of photocatalytic materials to change the surface properties and the electronic distribution [15]; doping ions to separate the electron hole pairs, to form the impurity energy levels, and more active centers [16,17,18,19]; compounding more than two different semiconductors to promote the separation of electron hole pairs [20]; controlling morphology and size to adjust the surface properties and the quantum size effect [21,22]. Therefore, in order to further improve the photocatalytic activity of Bi_2_WO_6_, it was coupled with the wide band gap semiconductor TiO_2_ photocatalyst to form a heterojunction nanocomposite [23,24]. The synergistic effect between TiO_2_ and Bi_2_WO_6_ can improve the light adsorption capacity and the separation efficiency of photogenerated electron hole pairs, prolonging the life of photogenerated carriers and enabling them to participate more in the photocatalytic reaction. In addition, the combination also effectively avoids the agglomeration of nano-titanium dioxide.

However, the recovery and separation of photocatalyst became the problem of wastewater treatment by photocatalysis. At present, there is little research on magnetic Bi_2_WO_6_/TiO_2_ [25], and it still needs two sets of devices, namely, a photocatalysis reactor and a catalyst reclaimer. To establish a photocatalytic reactor with high mass transfer efficiency, simple operation, and no catalyst separation is significant for the application of photocatalysis in the field of wastewater treatment.

In this study, Bi_2_WO_6_/TiO_2_ was synthesized by a simple microwave solvothermal method. Then, Bi_2_WO_6_/TiO_2_ was magnetized by the ammonia-iron double-drop method. Furthermore, the photocatalytic activity of Bi_2_WO_6_/TiO_2_/Fe_3_O_4_ (mBT) composite photocatalyst was evaluated by the novel mBT-MPR visible light catalytic system with 5.5 W, which was proposed to treat 220 mL of RhB simulated wastewater. The mBT-MPR visible light photocatalysis system is a green and efficient treatment technology for organic pollutants in water with simple operation, low energy consumption, and no need for catalyst separation.

## 2. Materials and Methods

### 2.1. Reagents and Instruments

The main analytically pure reagents include: Bi(NO_3_)_3_·5H_2_O, Na_2_WO_4_·2H_2_O, HNO_3_, CH_3_COOH, FeCl_3_·6H_2_O, FeCl_2_·4H_2_O, NH_3_·H_2_O, absolute ethanol, RhB (C_28_H_31_ClN_2_O_3_), and etc. Nanometer titanium dioxide (P25, Degussa AG, average particle size 21 nm, BET surface area 50 ± 15 m^2^/g, anatase/rutile = 80:20) was purchased from Lijie Chemical Co., Ltd., Shaoxing, China. The annular focusing microwave synthesizer (Discover, CEM, Matthews, NC, USA) was used to prepare the photocatalysts by the microwave solvothermal method. The concentration of RhB was analyzed by an ultraviolet-visible intelligent multiparameter tester (LH-3BA, Beijing Lianhua Technology, Peking, China). The scanning electron microscope-energy spectrometer (Regulus 8100, Hitachi, Tokyo, Japan) and field emission transmission electron microscope (Tecnai G2 F20, FEI, Columbia, MD, USA) were employed to analyze photocatalyst morphology and element composition. An X-ray diffractometer (D8 ADVANCE, Bruker, Salbruken, Germany) was used to observe the crystal structure of mBT. Elements and valence states were detected by X-ray photoelectron spectroscopy (ESCALAB 250XI, Thermo, Waltham, MA, USA).

### 2.2. Preparation of mBT Photocatalyst

Bi_2_WO_6_, a certain amount of Bi (NO_3_)_3_·5H_2_O (5.4328 g) was dissolved into 10 mL of a 1 mol/L HNO_3_ or a 17.5 mol/L CH_3_COOH solution, and then stirred for 1 h until the white suspension became colorless, this was marked as solution A. Na_2_WO_4_·2H_2_O (1.8472 g) was dissolved in 5 mL of deionized water and marked as solution B. Then, solution B was added into solution A slowly by a rubber tip, stirred for 1 h and marked as solution C, putting into the microwave synthesizer at 160 °C for 1 h reaction. Finally, the white sediment was cleaned with deionized water, followed by absolute ethanol more than three times.

Bi_2_WO_6_/TiO_2_ (BT), the P25 (0.4473 g) was dispersed in 20 mL of an aqueous solution for 2 min by ultrasonic, and then added into solution C described above ultrasonic dispersed for 5 min, putting into the microwave synthesizer at 160 °C for 1 h. The cleaning process of white sediment was the same as mentioned above.

**Figure 1:**
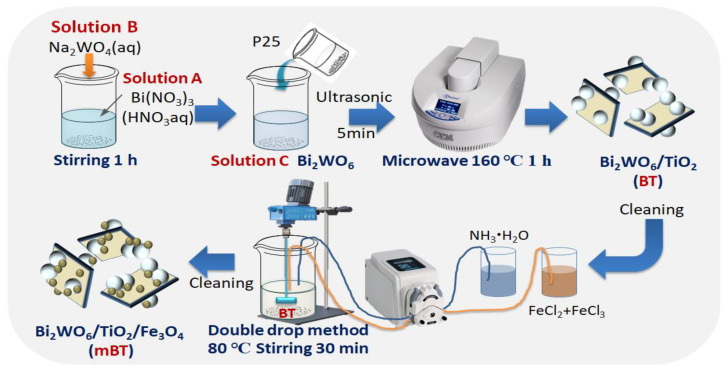
The schematic diagram of the synthesis of mBT and the ammonia iron double drop method.

Bi_2_WO_6_/TiO_2_/Fe_3_O_4_ (mBT), the synthesis process of mBT is shown in Figure 1. A certain amount of BT was magnetized by the ammonia-iron double-drop method. FeCl_2_·4H_2_O (0.5572 g) and FeCl_3_·6H_2_O (1.5148 g) (molar ratio Fe^2+^:Fe^3+^ = 1:2) were dissolved in 2.3 mL of deionized water, heated at 70 °C and mechanically stirred for 20 min to obtain a 3.75 mol/L iron ion solution. Then 2.3mL of the iron ion solution and ammonia (2.3 mL 13.3 mol/L) were simultaneously dropped into the above uniformly dispersed 40 mL Bi_2_WO_6_/TiO_2_ aqueous solution by the peristaltic pump, and stirred for 30 min at 80 °C. Finally, the obtained mixed solution was heated and stirred for 30 min, and cross cleaned by deionized water and anhydrous ethanol three times. 

### 2.3. mBT-MPR Visible Light Photocatalytic System Experiment

A tubular magnetic field-controlled photocatalytic reactor (MPR) was established, as referred to in the previous works [26]. The RhB simulated wastewater (220 mL) containing a certain amount of mBT photocatalyst was sent into the bottom of the MPR photoreactor by a peristaltic pump. 

Firstly, a dark reaction lasting 20 min was carried out to demonstrate the adsorption equilibrium between the catalyst and RhB pollutant molecules. Then, the LED light belt (5.5 W) was turned on to perform photocatalytic degradation of Rhodamine B (RhB). Samples shall be taken every 10 min for 1 h degradation. Finally, the concentration of RhB in effluent was obtained by measuring the absorbance at 550 nm wavelength with an ultraviolet-visible spectrophotometer.

## 3. Results and Discussion

### 3.1. The Characterization of mBT 

#### 3.1.1. Surface Morphology and Internal Structure

**Figure 2:**
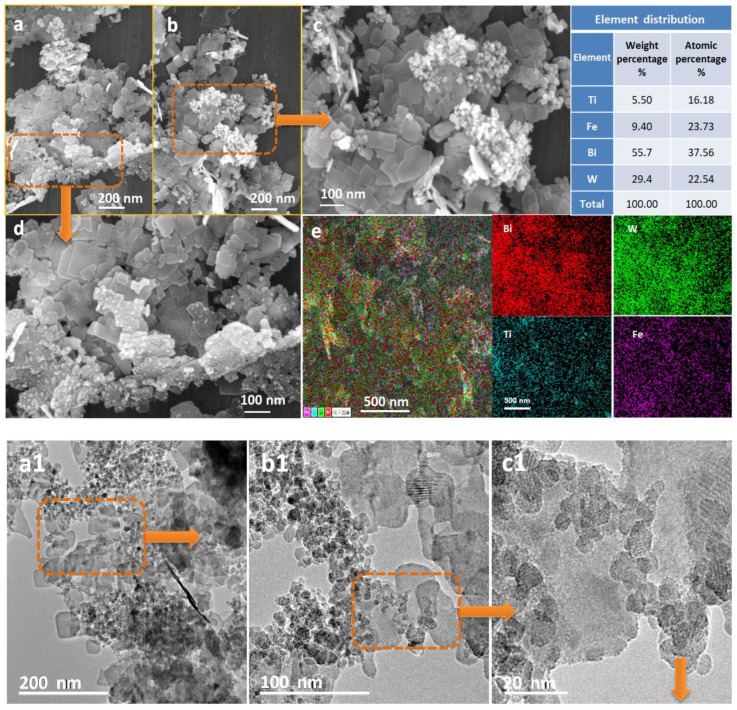

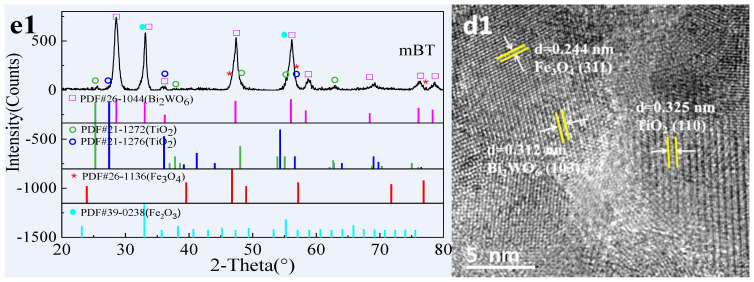
The SEM image (**a**–**d**) and EDS mapping of mBT (**e**); the HRTEM image (**a1**–**c1**) and the lattice marking (**d1**) of mBT; and the XRD image of mBT (**e1**).

The surface morphology, element composition, and internal structure of mBT were investigated by SEM, EDS, and HRTEM as shown in Figure 2. 

Bi_2_WO_6_ was assembled from a large number of irregular two-dimensional nano-sheets with a length of 20 nm to 120 nm, and TiO_2_ (25 nm) and Fe_3_O_4_ (6–12 nm) particles were distributed on its surface. Some particles were distributed on the two-dimensional Bi_2_WO_6_ surface as fish eggs due to magnetism, as shown in Figure 2c. Some particles combined well with Bi_2_WO_6_ and formed a stable heterojunction, as shown in Figure 2d. The element layering image of the composite was shown in Figure 2e, which was composed of Bi, W, Ti, and Fe elements; no other elements were detected, which was consistent with the characterization results of X-ray diffraction and X-ray photoelectron spectroscopy, further proving the successful preparation of mBT. 

Analyzing the HRTEM images of Figure 2(a1–c1), TiO_2_ and Fe_3_O_4_ nanoparticles were embedded in Bi_2_WO_6_ nanosheets. The Bi_2_WO_6_ lattice, TiO_2_ lattice, and Fe_3_O_4_ lattice of this composite material are shown in Figure 2(d1). The crystal structure of the prepared samples was further understood by an X-ray diffractometer. The strong and sharp diffraction peaks showed that the prepared samples had high crystallinity. The X-ray diffraction spectrum of the composite material mBT is shown in Figure 2(e1). This composite material, mBT, havs diffraction peaks at 2θ = 28.6, 33.0, 47.3, and 56.0°, which correspond to the crystal planes of (103), (200), (220), and (303) of Bi_2_WO_6_ (JCPDS26-1044). The diffraction peaks at 2θ = 25.3 27.4, 36.1, 37.8, 48.0, 54.3, and 62.7° correspond to the crystal planes of (101), (110), (101), (004), (200), (211), and (204) of TiO_2_ (JCPDS21-1276) and (JCPDS21-1272), respectively. The diffraction peaks at 2θ= 46.77, 57.18, and 76.88° correspond to the crystal planes of (311), (440), and (511) of Fe_3_O_4_ (JCPDS26-1136), indicating the successful preparation of mBT photocatalyst. 

#### 3.1.2. Elements and Valence States Analysis

**Figure 3:**
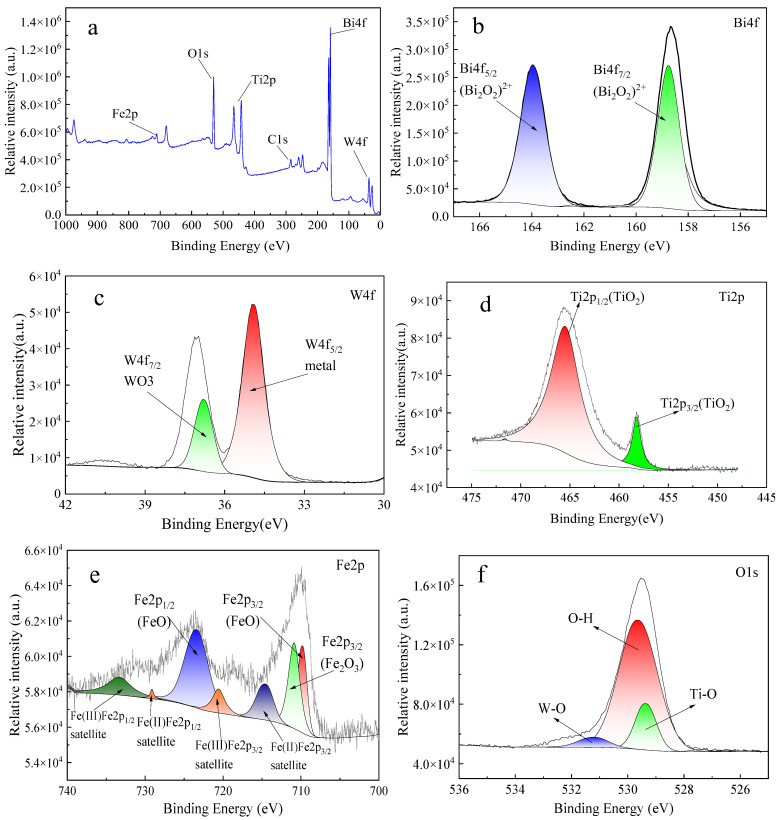
XPS total spectrum image of mBT (**a**) and the special spectrum of Bi4f (**b**), W4f (**c**), Ti2p (**d**), Fe2p (**e**), and O1s (**f**).

The chemical composition and electronic state of the mBT visible light photocatalyst were analyzed by XPS. As the results shown in Figure 3a indicate, the full spectrum indicates the material is mainly composed of bismuth, tungsten, oxygen, titanium, and iron. The presence of a C1S peak may be due to carbon dioxide in the air. Figure 3b–f shows the high-resolution XPS spectra of each element of the material. The peaks at 158.73 eV and 163.96 eV (Figure 3b) are attributed to Bi4f_7/2_ and Bi4f_5/2_, respectively, which are the characteristics of Bi^3+^ ions in Bi_2_WO_6_. The binding energies of the two peaks are 34.89 eV and 36.78 eV, respectively, (Figure 3c), which belong to W4f_5/2_ and W4f_7/2_, indicating that W exists in the form of +6 valence in this material. In Figure 3d, the two peaks of 458.22 and 465.47 eV correspond to Ti2p_3/2_ and Ti2p_1/2_, respectively. As shown in Figure 3e, the two peaks at 710.88 and 723.4 eV correspond to Fe2p_3/2_ and Fe2p_1/2_, respectively. The existing forms of Fe are FeO and Fe_2_O_3_, indicating the existence of Fe^2+^ and Fe^3+^ in the material and the synthesis of Fe_3_O_4_. As shown in Figure 3f, the binding energies of 529.63 eV and 529.35 eV correspond to the O-H bond in Bi_2_WO_6_ and the Ti-O bond in TiO_2_, and 531.17 eV corresponds to the W-O bond in (WO4) ^2−^ [27,28,29].

### 3.2. Screening of Synthesis Methods for mBT

**Figure 4:**
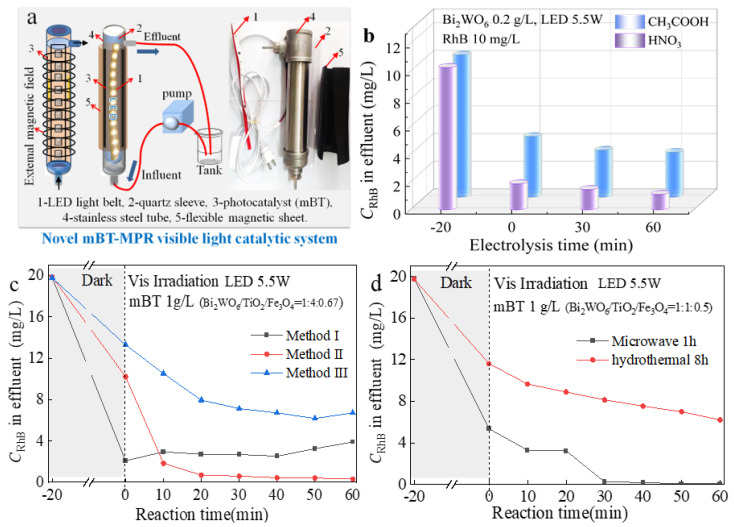
Schematic diagram of the mBT−MPR system (**a**), RhB degradation effect using Bi_2_WO_6_ in different solvents (**b**), using mBT by three synthesis methods (**c**), and two heating modes (**d**).

The mBT-MPR photocatalysis system shown in Figure 4a was established to treat RhB simulated wastewater to evaluate the photodegradation performance of visible light catalyst. As shown in Figure 4a, a visible light LED light belt (warm white light 3000–3500 k, 50 cm, 5.5 W, Yuexinguang Lighting, Shenzhen, China) was embedded in a quartz sleeve (Φ2.8 × 21 cm) as inner light source. The stainless-steel tube shell (Φ3.2 cm × 22 cm; effective volume 170 mL) was covered with a flexible magnetic sheet. The RhB solution was photodegraded by passing through the gap between the stainless-steel shell and the quartz sleeve. 

Firstly, the influence of the solvent on the photocatalytic activity for Bi_2_WO_6_ was investigated in Figure 4b. The preparation process of Bi_2_WO_6_ is described in Section 2.2. The concentrations of CH_3_COOH and HNO_3_ were 1 mol/L and 17.5 mol/L (analytically pure), respectively. The adsorbed and photodegraded effect of Bi_2_WO_6_ prepared with different solvents was evaluated by the concentration of effluent. The prepared Bi_2_WO_6_ has a larger surface area using HNO_3_ as solvent than CH_3_COOH due to the more oxidative nature of the solvent, which results in a higher removal rate of RhB.

The three synthesis methods mentioned in Figure 4c are as follows. Method I: the dispersion of Fe_3_O_4_ (0.87 g), P25 (1.7891 g) and 50 mL of deionized water; the solution B (Na_2_WO_4_) was dropped into the solution A (Bi(NO_3_)_3_) to obtain mBT by microwave synthesis. Method II: P25 dispersion was added to solution C (Bi_2_WO_6_) to obtain BT by microwave synthesis. Then, the mBT was prepared using the ammonia-iron double-drop method shown in Section 2.2. Method III: Bi_2_WO_6_ was prepared by the microwave solvothermal method. Then, the ultrasonic dispersion of Bi_2_WO_6_ and P25 was magnetized by the ammonia-iron double-drop method.

As the results shown in Figure 4c show, the mBT obtained by method II has the best degradation effect. The difference in RhB degradation effects caused by mBT prepared using three methods was reflected in the construction process of the Bi_2_WO_6_/TiO_2_ heterojunction. The presence of Fe_3_O_4_ in Method I will affect it. Method III cannot form heterojunction well, while Method II can better form it under the effects of microwave high temperature and high pressure, so it has a better photodegradation effect. 

The comparison between microwave synthesis for 1 h and hydrothermal reactor synthesis for 8 h was investigated by method II (Figure 4d). The results indicated the advantages of green and efficient microwave synthesis. The mBT catalyst was successfully synthesized by a microwave synthesis instrument with a short preparation time, referring to our previous research results [26]. 

### 3.3. Determination of the mBTcomposite Ratio and the Photocatalytic Mechanism

**Figure 5:**
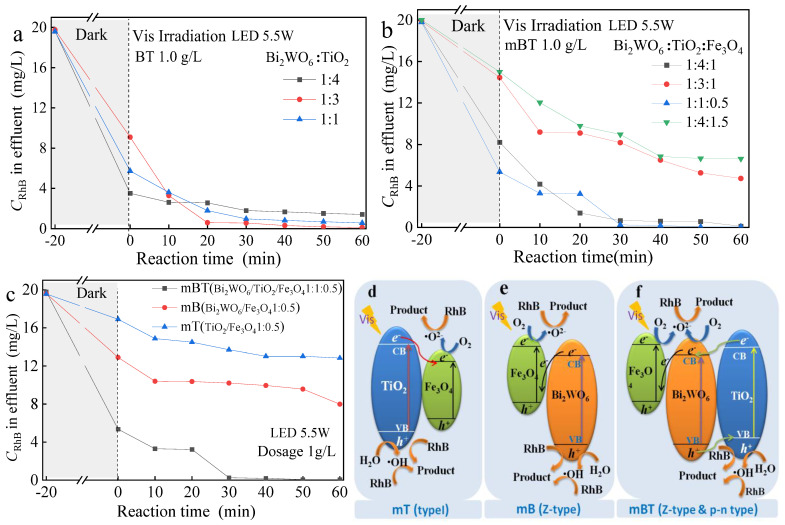
Degradation effect of RhB using Bi_2_WO_6_/TiO_2_ (BT) (**a**) and mBT (**b**) at different molar ratios and using three kinds of magnetic photocatalysts (**c**) with their photocatalytic mechanisms (**d**–**f**).

Different mole ratios of Bi_2_WO_6_/TiO_2_ will affect the heterojunction structure and result in a change in RhB degradation. In Figure 5a, properly increasing the TiO_2_ ratio to 1:3 is conducive to the formation of more heterojunctions, thus improving the photocatalytic activity of BT. However, a too-high ratio of TiO_2_ will reduce the visible light absorption efficiency of Bi_2_WO_6_, reducing the photocatalytic performance of BT. Therefore, a suitable ratio of Bi_2_WO_6_/TiO_2_ will promote better transmission of electrons and holes by the heterojunction of the two. 

Analyzing the results in Figure 5a,b, the presence of Fe_3_O_4_ was investigated to affsee how it affected the photocatalytic efficiency of mBT. When the molar ratio of Bi_2_WO_6_/TiO_2_/ Fe_3_O_4_ was 1:4:1, compared with the molar ratio of Bi_2_WO_6_/TiO_2_, the dark reaction adsorption decreased by about 4.7 mg/L and the photocatalytic degradation increased by 6 mg/L. This demonstrated that the presence of Fe_3_O_4_ will affect the adsorption by reducing the surface of Bi_2_WO_6_, but it inhibits the recombination of photogenerated carriers through participating heterojunction and improves photocatalytic efficiency. Continued increase in the proportion of Fe_3_O_4_ to 1:3:1 and 1:4:1.5, both the adsorption and the photocatalytic effect were obviously affected. It indicated that Fe_3_O_4_ would occupy the adsorption site and the photocatalytic active site. When the molar ratio of Bi_2_WO_6_/TiO_2_/ Fe_3_O_4_ was 1:1:0.5, compared to a 1:1 molar ratio for Bi_2_WO_6_/TiO_2_, the presence of Fe_3_O_4_ did not have an obvious impact on the adsorption and photocatalytic efficiency. Considering the coupling effect of adsorption and photodegradation, the synthesis scheme of mBT was adopted with a molar ratio of 1:1:0.5. After 40 min of light, the RhB degradation rate achieved 99.7%. 

In order to verify the degradation mechanism of photocatalysts with different heterojunction types, the experiments shown in Figure 5c were carried out. The degradation effect of RhB was significantly improved using mBT compared with mB and mT. That demonstrated more heterojunction formed when Bi_2_WO_6_ contacted Fe_3_O_4_ and TiO_2_ at the same time. The heterojunctions can greatly inhibit the recombination of photogenerated carriers, improving the photocatalytic performance of the photocatalyst.

The formation process of heterojunction and the principle of promoting RhB degradation are shown in Figure 5d–f. The heterojunction type of mT is the traditional type I shown in Figure 5d. The electrons transfer from the VB (valence band) to the CB (conduction band), resulting in electron hole pairs. The electrons of CB transfer from TiO_2_ to Fe_3_O_4_, producing ∙O^2−^, while the holes accumulate in the VB of TiO_2_ to generate a large amount of ∙OH. The generated ∙O^2−^ and ∙OH are the main oxidants that degrade RhB molecules [30].

As the process shown in Figure 5e shows, the heterojunction between Fe_3_O_4_ and Bi_2_WO_6_ is Z-type in Figure 5e,f. For two kinds of semiconductors, the heterojunction between TiO_2_ and Bi_2_WO_6_ belongs to p-n type according to references [31,32]. Bi_2_WO_6_ and Fe_3_O_4_ first absorb visible light energy to produce abundant of *e*^−^ in the CB of Bi_2_WO_6_. Then, the electrons migrate to VB in Fe_3_O_4_ and recombine with *h*^+^, promoting the effective separation of electron hole pairs in Bi_2_WO_6_ or Fe_3_O_4_. The *e*^−^ of Fe_3_O_4_ returns O_2_ to ∙O^2−^, the *h*^+^ of Bi_2_WO_6_ converts to ∙OH [33].

The RhB photodegradation mechanism by mBT was investigated and shown in Figure 5f. After Bi_2_WO_6_ and TiO_2_ absorb visible light energy, *e*^−^ at the CB of TiO_2_ is easily transferred to the CB of Bi_2_WO_6_, so that O_2_ is reduced by *e*^−^ to produce ∙O^2−^. At the same time, *h*^+^ frm the VB of Bi_2_WO_6_ migrates to the CB of TiO_2_, and *h*^+^ reacts with H_2_O to produce ∙OH, ∙O^2−^ and ∙OH degrade RhB molecules into an intermediate product and *h*^+^ can also directly oxidize RhB molecules. The recombination rate of electrons and holes is greatly reduced, thereby improving the photocatalytic performance of the photocatalyst [33,34,35]. The N-ethyl group on the original RhB molecule is continuously attacked by the active radicals (∙OH, *h*^+^, and ∙O^2−^), the electronic delocalization of N-ethyl became weaker, the ethyl group detaches from different parts of RhB, and the opening-ring process continues with the continuous attack by active radicals [36]. 

### 3.4. The analysis of Cyclic Stability and Energy Consumption

**Figure 6:**
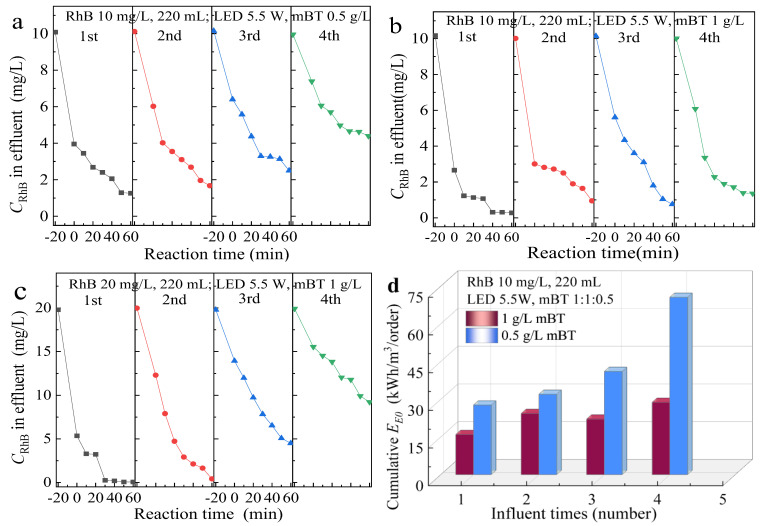
Comparison of the photodegradation stability of mBT with different dosages ((**a**) vs. (**b**)) and initial concentrations of RhB ((**b**) vs. (**c**)), and the accumulative electric energy consumption of the mBT−MPR system under different mBT dosages (**d**).

As shown in Figure 6a–c, the mBT-MPR visible light catalytic system (LED 5.5 W) was used to treat 220 mL of RhB simulated wastewater with an initial concentration of 10 mg/L or 20 mg/L.

In Figure 6a, when the dosage of mBT is 0.5 g/L, the 10 m/L RhB wastewater is treated four times continuously, and the removal stability of the effluent is not satisfactory. It can be seen from the data in Figure 6a that the adsorption and photocatalytic performance of mBT decrease with the increase in use times. That indicates the amount of mBT is insufficient to fully degrade RhB at a concentration of 10 mg/L. Therefore, to investigate the relationship between the dosage of mBT and effluent stability, the dosage of mBT was increased to 1 g/L, as shown in Figure 6b. After two times of use, the adsorption effect of mBT obviously decreased, but the photocatalytic effect was improved, so that the stability of the effluent was better than in Figure 6a. 

In order to further verify the relationship between mBT dosage and RhB photodegradative stability, the initial concentration of RhB was increased to 20 mg/L in Figure 6c. When the dosage of mBT was 1 g/L, the stability of the effluent decreased significantly as the influent concentration increased to 20 mg/L. In conclusion, at the novel mBT-MPR visible light catalytic system, the dosage of catalyst mBT is closely related to the concentration of influent RhB. Only when the dosage of mBT is sufficient, can better effluent stability be achieved.

In this study, an LED light belt was used to simulate visible light. To calculate the electric energy consumption, a parameter of electrical energy per order (*E_EO_*, kWh/m^3^/order) can be defined as the electrical energy (kWh) for 90% removal by one order of magnitude per m^3^ of contaminated water. It combines light intensity, residence time, and volume with pollutant removal efficiency into a single measurement method [37,38,39,40]. The *E_EO_* value is calculated using this expression:

(1) $$E_{EO}=\frac{Pt}{V\log\left(C_{i}/C_{f}\right)}$$

where *P* is the power of the LED light belt (kW, 0.0055 kW in this study), t is the irradiation time (H), *V* is the volume of simulated wastewater (m^3^, 220 mL in this study, 0.00022 m^3^), and *C_i_* and *C_f_* are the pollutant concentrations at the initial and final times (mg/L) respectively. Substitute the value to get *E_EO_*. As shown in Figure 6d, the cumulative electric energy consumption value of the mBT-MPR system was described under the different dosages of mBT (0.5 g/L or 1 g/L). When the initial concentration of RhB was 10 mg/L and the dosage of mBT was 1 g/L, the effluent was relatively stable, and the mean value of energy consumption of four times was 22.8 kWh/m^3^/order, which was obviously lower than that of 0.5 g/L mBT. According to the results of Table 1, the higher initial concentration and volume of organic pollutants will result in more difficulty with photocatalytic degradation, a longer degradation time, and a higher E_EO_ value. 

## 4. Conclusions

Magnetic composite visible light catalyst mBT was successfully prepared by the ammonia-iron double-drop and microwave solvothermal methods. The optimal molar ratio of Bi_2_WO_6_, TiO_2_, and Fe_3_O_4_ in the mBT photocatalyst was 1:1:0.5. The morphology results of mBT showed that TiO_2_ and Fe_3_O_4_ particles were loaded on the surface of Bi_2_WO_6_ sheets. Heterojunction structures of mBT are constructed by Fe_3_O_4_, TiO_2_, and Bi_2_WO_6_ in three parts, obviously improving the separation and transfer of photogenerated electron hole pairs at the heterojunction interface.

The novel mBT-MPR visible light catalytic system with 5.5 W was proposed to treat 220 mL of RhB simulated wastewater. The advantages of that system are simple operation, a good mass transfer effect, and the fact that no catalyst separation is required for the effluent. After 1 h of visible light irradiation, the average removal rate of RhB at an initial concentration of 10 mg/L for four times was 91.7%. 

The removal effect of RhB by the mBT-MPR photocatalytic system was mainly due to the coupling effect of adsorption and photocatalysis by mBT. The dosage of mBT is correlated with the concentration of influent RhB. To obtain stable effluent, a reasonable mBT dosage should be chosen to match the corresponding influent pollutant concentration.

## Figures and Tables

**Table 1:** Comparison of electrical energy consumption of different photocatalytic degradation systems.

Pollutants (*C_i_* mg/L; *V* mL)	Removal (%)	Photocatalyst (Dosage g/L)	Photocatalysis Conditions	Energy (kWh/m^3^/Order)	Ref.
Rhodamine B 10 mg/L, 220 mL	91.7% 60 min	Bi_2_WO_6_/TiO_2_/Fe_3_O_4_ 1 g/L	LED light belt 5.5 W	22.8 mean value	This study
Tetracycline 10 mg/L, 60 mL	68.4% 80 min	Ag_3_PO_4_ 0.17 g/L	Blue-LEDs 2.6 W	113	[38]
Tetracycline 30 mg/L, 60 mL	49.3% 80 min	Ag_3_PO_4_ 0.17 g/L	Blue-LEDs 2.8 W	212	[38]
Tetracycline 50 mg/L, 60 mL	45.2% 80 min	Ag_3_PO_4_ 0.17 g/L	Blue-LEDs 5.2 W	239	[38]
methylene blue 10 mg/L, 200 mL	47% 180 min	ZnCoFe LDH 0.1 g/L	LED lamps 100 W	5410	[39]
Reactive Black 5 20 mg/L, 1 L	89.9% 120 min	Fe_3_O_4_-WO_3_-APTES 0.2 g/L	LED lamp 15 W	34.7	[40]
